# Influence of Carbon Nanotube-Pretreatment on the Properties of Polydimethylsiloxane/Carbon Nanotube-Nanocomposites

**DOI:** 10.3390/polym13091355

**Published:** 2021-04-21

**Authors:** Astrid Diekmann, Marvin C. V. Omelan, Ulrich Giese

**Affiliations:** Deutsches Institut für Kautschuktechnologie e. V., Eupener Straße 33, 30519 Hannover, Germany; astrid.diekmann@dikautschuk.de (A.D.); marvin.omelan@dikautschuk.de (M.C.V.O.)

**Keywords:** dispersion, filler–filler interactions, functionalization, polydimethylsiloxane, carbon nanotubes

## Abstract

Incorporating nanofillers into elastomers leads to composites with an enormous potential regarding their properties. Unfortunately, nanofillers tend to form agglomerates inhibiting adequate filler dispersion. Therefore, different carbon nanotube (CNT) pretreatment methods were analyzed in this study to enhance the filler dispersion in polydimethylsiloxane (PDMS)/CNT-composites. By pre-dispersing CNTs in solvents an increase in electrical conductivity could be observed within the sequence of tetrahydrofuran (THF) > acetone > chloroform. Optimization of the pre-dispersion step results in an AC conductivity of 3.2 × 10^−4^ S/cm at 1 Hz and 0.5 wt.% of CNTs and the electrical percolation threshold is decreased to 0.1 wt.% of CNTs. Optimum parameters imply the use of an ultrasonic finger for 60 min in THF. However, solvent residues cause a softening effect deteriorating the mechanical performance of these composites. Concerning the pretreatment of CNTs by physical functionalization, the use of surfactants (sodium dodecylbenzenesulfonate (SDBS) and polyoxyethylene lauryl ether (“Brij35”)) leads to no improvement, neither in electrical conductivity nor in mechanical properties. Chemical functionalization enhances the compatibility of PDMS and CNT but damages the carbon nanotubes due to the oxidation process so that the improvement in conductivity and reinforcement is superimposed by the CNT damage even for mild oxidation conditions.

## 1. Introduction

Elastomer-composites consist of a crosslinked rubber matrix and several additives to reach the performance needs and to fulfill processing, technical, and economical requirements. Filler particles in the nanoscale are incorporated to enhance the properties of the composite like mechanical and dynamic-mechanical properties, electrical conductivity, or chemical resistance, for instance. Carbon nanotubes (CNTs) represent such a filler type in the nanoscale which makes their application in elastomer compounds extremely beneficial. CNTs consist of rolled up graphene sheets leading to a high specific surface area and a high aspect ratio [1] implying improvements regarding the reinforcement of elastomers. This has been proven successfully for several times [2,3,4,5,6,7]. In addition, CNTs possess a high electrical conductivity in their pristine form due to their sp^2^-hybridized structure [1,8]. By using multiwall carbon nanotubes (MWCNTs) in a polymeric matrix, a conductivity level of up to 4000 S/cm can be attained [3]. Though, nanofillers exhibit high particle–particle interactions due to van der Waals forces and therefore tend to form agglomerates [9,10]. It is particularly challenging when incorporating CNTs into a polymer matrix as the agglomeration behavior hinders the formation of a homogeneous filler dispersion in the polymer matrix. To take benefit of the enormous potential of carbon nanotubes it is inevitable to break up the agglomerates to disperse the carbon nanotubes adequately and to offer a high contact surface area for optimal polymer–filler interactions. Furthermore, adequate filler distribution and dispersion enable to reduce the filler amount which is favorable to decrease the density of composites and to lower the costs, especially regarding potentially high reinforcing nano-fillers like CNTs.

To realize high filler dispersion, it is mandatory to optimize the processing step, as applying well-balanced shear forces enables to break up agglomerates during the compounding step already [11]. An additional agglomerate breakage can be achieved by separating the carbon nanotubes not only during the processing step but prior to this. Therefore, the particle–particle interactions have to be overcome which can be realized by a pre-dispersion step where the CNTs are dispersed in a solvent before mixing, for instance. In addition, enhancing polymer–filler interactions to overcome filler–filler interactions and to improve the bonding between the elastomer and the filler particles in consequence is another approach in increasing the filler dispersion. This can be realized for example by functionalization of the filler surface in order to change their surface polarity and to enhance compatibility. There are several attempts on increasing CNT dispersion in elastomeric matrices concerning compounding techniques, pre-dispersion, and functionalization reported in the literature up to now, see for example [10,11,12,13,14,15,16,17,18,19]. Concerning the incorporation of CNTs into silicone rubber, increased reinforcement and electrical and thermal conductivity are reported [20,21,22], especially dealing with hybrid materials [23]. Pre-dispersion and functionalization methods are described as well [20,24]. Regarding polydimethylsiloxane (PDMS)-composites filled with CNTs in particular, only minor studies have been made. These composites are of special interest concerning their electrical conductivity, as electrically conductive elastomers are used for shielding purposes, as strain sensors or electrodes [25,26,27,28,29]. The combination of an insulating silicone matrix and CNTs as an electrically conductive filler leads to overall conductivity of the material with simultaneous prevention of electrical arcs between different components [21]. Dealing with PDMS/CNT-composites, high reinforcement values and decreased electrical resistances are observed as well [20,28,30].

To overcome difficulties in mixing of low viscous PDMS with CNT a planetary mixing strategy with optimized shear forces is used [11].

This study underlies the preparation of extremely soft and electrically conductive elastomers to potentially be used as electrode material in medical applications [11]. The approach is to improve the dispersion of CNTs in PDMS by applying an additional pretreatment step to overcome filler–filler interactions and to separate the carbon nanotubes before the mixing process, already. This is realized by applying an appropriate pre-dispersion step using different solvents and by modifying the CNT surface via functionalization. At this, the pre-dispersion leads to a reduction of filler–filler interactions whereas the functionalization enhances polymer–filler interactions due to polarity equalization and the application of functional groups and therefore reduces filler–filler interactions in consequence.

## 2. Materials and Methods

### 2.1. Materials

A two-component, addition cured polydimethylsiloxane (Sylgard 184, Dow Corning Inc., Wiesbaden, Germany) was used as silicone rubber, where a ratio of 10:1 of base to curing agent is recommended. The electrically conductive filler particles were multiwall CNTs (Nanocyl7000, Nanocyl^TM^, Sambreville, Belgium) with a specific surface of 270 m^2^/g [6].

For realizing the pre-dispersion step, tetrahydrofuran (THF), acetone, and chloroform (CHCl_3_) were chosen as solvents. Oleic acid, sodium dodecylbenzenesulfonate (SDBS), and polyoxyethylene lauryl ether (“Brij35”) were used for physical functionalization. Chemical functionalization was realized based on different concepts which are described in Table 1 in detail.

### 2.2. Pretreatment and Compounding

Ultrasonic treatment was performed using an ultrasonic bath RK 255 H (Co. Bandelin Sonorex, Berlin, Germany) and an ultrasonic finger UP200S (Co. Hielscher, Teltow, Germany) with a mode of 0.5, an amplitude of 80%, and a power density of 300 W/cm^2^. The ultrasonic lengths were varied throughout the experiments in between 30 and 120 min.

For the pre-dispersion step CNTs were sonicated in 500 mL of solvent, concentrated in a rotary evaporator, and added to the PDMS base afterwards. Physical functionalization was realized performing the sonication by adding oleic acid or surfactants (SDBS, “Brij35”) to 250 mL THF or ethanol. To enhance the evaporation process, aqueous solvent mixtures of THF/H_2_O at a ratio of 5:1 were used. Chemical functionalization was performed using the reaction conditions listed in Table 1 and the cited literature therein. The oxidation and reduction processes were carried out in a three-necked flask whereas the final functionalization proceeded in a polypropylene flask as it is inert against trichlorosilane.

For the preparation of the compounds a twin tool planetary mixer (LPV 1A40, PC Laborsystem, Magden, Switzerland) was used as the viscosity of PDMS contradicts the use of conventional internal mixers. An integrated bell jar offers the possibility to evaporate the mixture (−1000 mbar) while simultaneously stirring. The mixing procedure was based on [11] consisting of a 30 min degassing step and the compounding step for 10 min at 300 rpm. Solvent residues were evaporated for three hours using a heated silicon sleeve (45 °C) after the compounding step. The subsequent homogenizing of the curing agent (second silicon-component) was performed at a rotor speed of 150 rpm for 90 s. Finally, the compound was evaporated for another 60 min.

The vulcanization was performed at 150 °C in a vacuum heating press (KV 234.00, J. Wickert & Söhne, Landau, Germany) using a pressure of 280 bar for 14 min and monitored by a rubber process analyzer (Monsanto RPA 2000, Columbia City, USA) at 150 °C.

### 2.3. Characterization

Dielectric spectroscopy (broadband analyzer BDS 40, Novocontrol GmbH, Montabaur, Germany) was performed in a frequency range of 0.1 Hz to 10 MHz at room temperature on crosslinked cylinder samples (Ø = 20 mm, d = 2 mm). To ensure contact, the samples were coated with gold on both sides (Polaron SC7640, Thermo VG Scientific, Germany).

Tensile tests were carried out on S2 specimen using a universal testing device (Zwick 1445, Co. Zwick Roell GmbH & Co. KG, Ulm, Germany) with 0.5 N pre-load and 200 mm/min tensile speed. The illustrated values correspond to an average of 5–7 measurements each, using median curves for further analyses.

Shore A hardness was estimated on a Zwick digitest at room temperature on samples with 6 mm of thickness. The values correspond to mean values of five measurements.

The transmission electron microscopic images (TEM) were taken using a LIBRA 120 microscope (Co. Carl Zeiss AG, Oberkochen, Germany) with an acceleration voltage of 120 kV. Thin sections of the material were cut using a cryo ultramicrotome at a temperature of −140 °C (diamond knife, diatome 35°) and placed on a 600 mesh copper grid.

Thermal gravimetric analyses (TGA) on functionalized samples were performed on a TA-Instruments TGA 2950CE-Hi-Res (New Castle, DE, USA) in a temperature range of 25–950 °C using nitrogen as purging gas to prevent further oxidation. Samples were heated for 24 h at 150 °C to evaporate potential solvent residues.

In order to investigate functional groups and surface defects of the CNTs, Raman-spectra (Bruker Senterra Raman Microscope, Billerica, MA, USA) were recorded using the following parameters: Acq. 10 s × 5 loops, obj. 50×, λ = 532 nm, *P* = 2 mW, Ap: 50 × 1000 µm, Res: 3–5 cm^−1^, Range: 50–1555 cm^−1^, *T* = 21.8 °C, *H* = 42%.

X-ray photoelectron spectroscopy measurements (XPS) were carried out on a spectrometer of Leybold-Heraeus GmbH (Köln, Germany). Samples were prepared on an indium-specimen holder and evacuated for at least one hour. Analyses were performed using Al-Kα-radiation (*hv* = 1486.6 eV, *HV* = 7 kV, *I*-Anode = 30 mA) under ultra-high vacuum (10–8 mbar) at room temperature and light exclusion using a hemispheric analyzer (Ø = 100 mm, *PMV* = 2.7 kV, Δ*E* = constant).

## 3. Results

### 3.1. Pre-Dispersion

**Figure 1:**
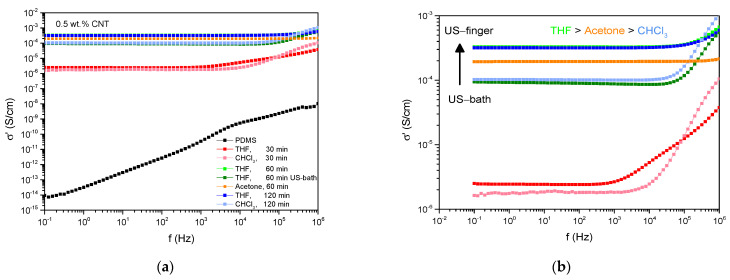
Dielectric results in dependency of ultrasonic terms (**a**) all results and (**b**) pre-dispersion results in detail.

As an appropriate pre-dispersion step enables to break up CNT agglomerates due to the decrease of particle–particle interactions, different pre-dispersion approaches were investigated in this study. After the pre-dispersion step, the subsequent compounding was performed at optimum conditions according to [11] to combine the enhancement in filler dispersion by pre-dispersion and by the agglomerate breakage arising from shear forces. To realize the pre-dispersion step, ultrasonication was applied using different techniques, conditions, and solvents. Figure 1 displays the electrical conductivity against frequency depending on the ultrasonic terms in PDMS/CNT compounds with 0.5 wt.% of CNTs. The composite filled with 0.5 wt.% CNT without pretreatment is insulating itself. By implementing a pre-dispersion step, an enormous increase in conductivity can be observed for all samples ending up in percolated systems. As the CNT amount remains unchanged, this indicates decreased filler–filler interactions and improved agglomerate break-up due to the pre-dispersion step in consequence. Concurrent to this, decreased filler–filler interactions increase the contact area between the polymer and the filler which increases CNT-PDMS bonding options and therefore polymer–filler interactions.

Dealing with an ultrasonic time of 30 min in THF leads to an increase of conductivity of eight decades at 1 Hz. By further increasing the duration up to 60 min an additional increase of conductivity up to 3.2 × 10^−4^ S/cm can be observed with no substantial increase at longer sonication terms.

Comparing the ultrasonic techniques, the use of an ultrasonic finger dominates the ultrasonic bath. Using the ultrasonic finger instead of the bath, when dispersing CNTs for 60 min in THF, leads to an increase of conductivity by three times. This may be attributed to the re-agglomeration effects when using the ultrasonic bath as the ultrasonic finger transmits its energy in a more restricted range. Regarding the kind of solvent, the highest conductivity values are realized using THF which implies improved agglomerate break-up and dispersion. Overall, THF performs better than acetone than chloroform ending up in the tendency of THF > acetone > chloroform which is in coherence with the eluting order of these solvents. As the order of polarity corresponds to the order of dielectric conductivity of the solvents, it can be assumed that solvent residues contribute to the conductivity as well.

**Figure 2:**
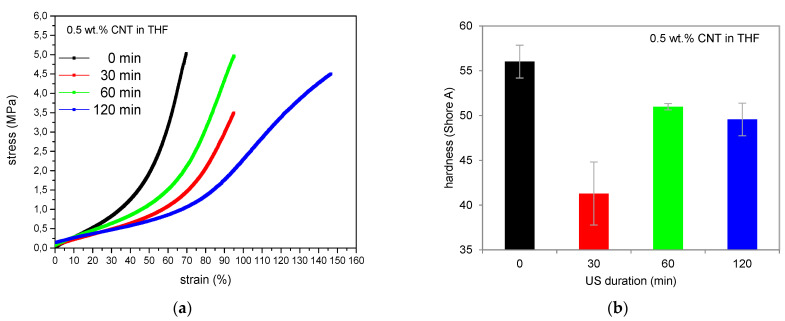
Mechanical behavior of polydimethylsiloxane/carbon nanotube (PDMS/CNT)-composites with pre-dispersion in THF, (**a**) stress-strain experiments and (**b**) Shore A hardness.

Therefore, additional mechanical investigations were performed. Stress–strain experiments (Figure 2a) indicate a softening of all samples with pre-dispersed CNTs apparent by higher elongation at break values which confirms the assumption of solvent residues. A decrease in elastic modulus can be observed, which already occurs at low elongation values. This illustrates the absence of reinforcement, which is normally expected when incorporating nanofiller particles in an elastomeric matrix. The softening effect is likely caused by the adhesion of the solvent on the surface of the carbon nanotubes which leads to solvent deposition in the compound in consequence. Therefore, it will be necessary to control the content of solvent or avoid any solvents in the compound at least regarding further investigations.

Analyzing the stress–strain curves regarding the ultrasonic duration, maximum tensile strength can be observed at 60 min. Here, CNTs are dispersed best which partially compensates the softening effect. This is evident by an equivalent reinforcement compared to the initial level of the sample with untreated CNTs due to the enhanced dispersion and subsequent filler–polymer bonding.

Shore A hardness (Figure 2b) confirms the softening effect due to solvent residues as well, indicating decreased hardness. Best ultrasonic conditions are found at a sonication time of 60 min again.

**Figure 3:**
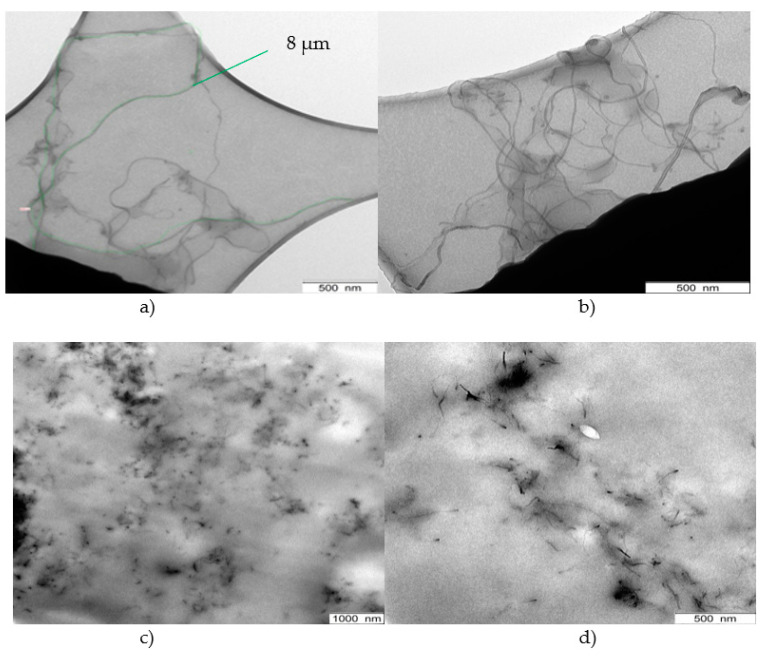
(**a**,**b**) TEM images of pre-dispersed CNTs (THF, 60 min) and (**c**,**d**) CNTs in PDMS (THF, 60 min).

Transmission electron microscopic (TEM) images are shown in Figure 3 in order to have a direct look on the filler agglomerates. Figure 3a,b illustrate CNTs dispersed in THF (60 min, US-finger) and applied on a copper grid directly. Effective agglomerate break-up can be observed here, where separated carbon nanotubes exist. These CNTs hold a length of up to 8 µm.

Images (Figure 3c,d) display the filler distribution in the PDMS compound with CNTs pre-dispersed at the same conditions. To ensure improved observation conditions, 1 wt.% of CNT were incorporated, which ensures to be above the percolation threshold [11]. The images demonstrate good filler dispersion in the PDMS matrix. Separated carbon nanotubes are still present and only minor clusters of small size are apparent.

**Figure 4:**
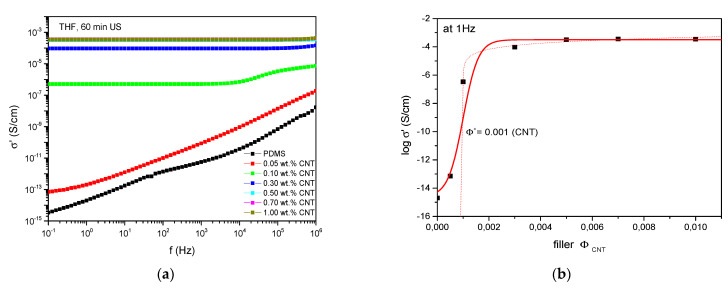
(**a**) Electrical conductivity depending on pretreated CNT concentration and (**b**) estimation of electrical percolation threshold.

Regarding the effectiveness of the pre-dispersion step, the percolation threshold was determined which is defined as the minimum filler content where a filler network is built and the electrical conductivity rises drastically. The formation of these conductive paths and therefore the percolation threshold depend on many factors such as the geometry, the intrinsic conductivity, and the state of dispersion of the corresponding nanofillers [36,37]. Dielectric measurements depending on the CNT content show an increase in conductivity with increasing CNT amount (Figure 4a). At a filler level of 0.1 wt.% a drastic increase in conductivity arises and at contents around 0.5 wt.% a plateau of the dielectric conductivity of 3.6 × 10^−4^ S/cm is reached where no further increase in conductivity occurs at higher CNT amounts. This plateau is reached at an extremely low filler amount, which again indicates ideal filler dispersion due to this pretreatment step, as the filler amount where the plateau in electrical conductivity exists is based on the distances between the filler particles. Therefore, the formation of conductive pathways is developed at a comparatively low concentration and is in good agreement with [38]. The electrical percolation threshold (Figure 4b) was calculated in accordance with [11] and resulted in 0.1 wt.% of CNTs at 1 Hz (corresponding to filler–polymer volume fraction of Φ* = 0.001). As the initial electrical percolation threshold without any pretreatment amounts to 0.9 wt.% of CNTs (Φ* = 0.009) [11], this is a distinct decrease in CNT concentration and stresses the aforementioned claims.

### 3.2. Functionalization

**Figure 5:**
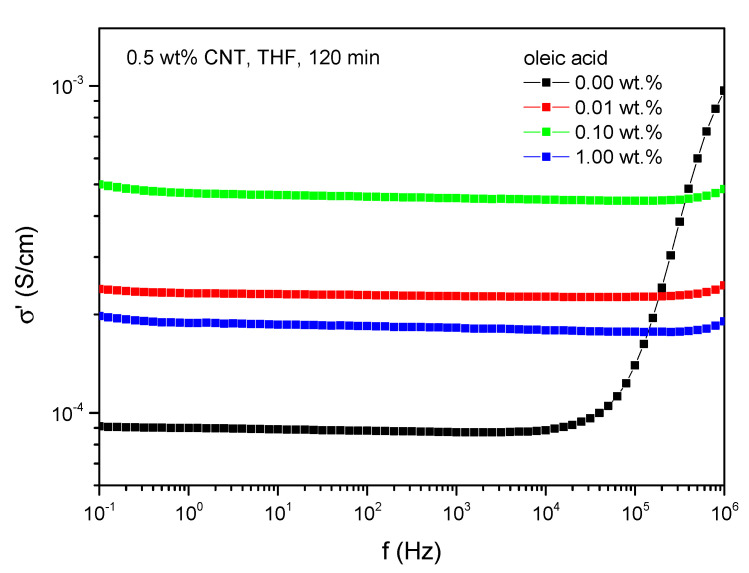
Dielectric conductivity depending on oleic acid concentration.

An additional concept of this study is to enhance polymer–filler interactions by increasing the compatibility of CNTs and PDMS as enhanced polymer–filler interactions superpose filler–filler interactions and therefore support agglomerate break-up. This can be realized by adding surface-active substances that are physically bonded on the filler surface and compensate polarity differences between filler and polymer. This procedure preserves the structure of the filler particles as the substances are bonded to the filler surface non-covalently which ensures to keep the properties of the filler. Oleic acid serves these criteria, which was added based on predispersion in THF. Figure 5 displays the dielectric conductivity of PDMS/CNT-composites with 0.5 wt.% CNT pretreated with sonication for 120 min with different concentrations of oleic acid. Adding oleic acid leads to a slight increase in conductivity, where the composites with 0.1 wt.% oleic acid and 0.5 wt.% CNTs reaches a conductivity of 5 × 10^−4^ S/cm at 1 Hz. Implementing higher concentrations of oleic acid decreases the conductivity, so that CNT dispersion is at an optimum using 0.1 wt.% of oleic acid.

The decrease in conductivity at high concentrations of oleic acid can be attributed to the formation of micelles of oleic acid. When adding high amounts of oleic acid, there is a spatial limitation of absorbance sites for oleic acid on the CNT surface [39]. Consequently, oleic acid molecules tend to enclose filler particles in their micelles. This ends up in an exemption of these filler particles regarding the formation of electrical paths and a subsequent decrease in conductivity.

**Figure 6:**
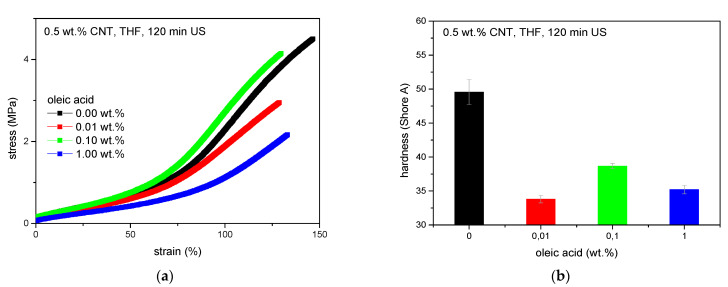
Results of mechanical experiments of composites pretreated with THF and oleic acid, (**a**) stress-strain curves and (**b**) Shore A hardness.

Mechanical experiments regarding the composites treated with oleic acid confirm best CNT dispersion when adding 0.1 wt.% oleic acid (Figure 6). In total, this method of pretreatment leads to a decrease in tensile strength and elongation at break compared to composites with merely pre-dispersed CNTs (Figure 6a). As the elongation at break decreases, softening due to solvent residues is not obvious. This implies that oleic acid covers the surface of the carbon nanotubes effectively which inhibits the adhesion of the solvent. In consequence, THF can be preferably removed in the vacuum processing step. Though, mechanical reinforcement cannot be observed, which is also approved by Shore A hardness (Figure 6b).

Further investigations on physical functionalization were performed using surfactants. Here, sodium dodecylbenzenesulfonate (SDBS) and polyoxyethylenelaurylether (“Brij35”) where chosen as they proved to stabilize CNT dispersions, where SDBS serves as an anionic surfactant and “Brij35” as a nonionic one [40]. Thereby, CNTs were pre-dispersed in a combination of the surfactant with ethanol or with a solution of THF/H_2_O (5:1), in order to facilitate the extraction of the solvent.

**Figure 7:**
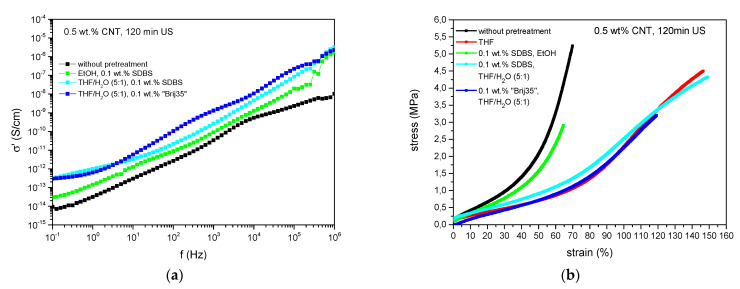
Composites with CNTs pretreated with surfactants, (**a**) dielectric conductivity and (**b**) stress–strain analyses.

**Figure 8:**
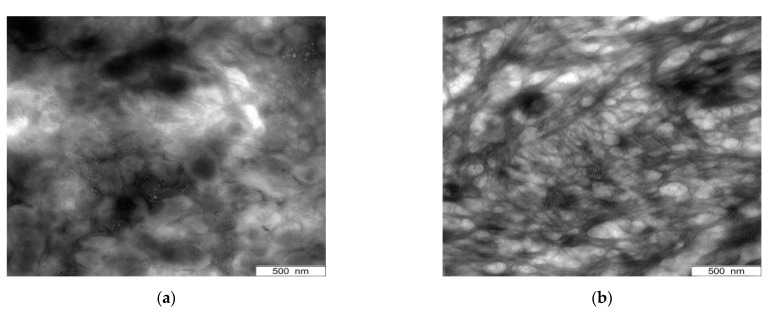
TEM images of CNTs pretreated with sodium dodecylbenzenesulfonate (SDBS). (**a**) for 10 min US and (**b**) 60 min US.

Dielectric results of the surfactant series demonstrate that there is no beneficial effect in this kind of pretreatment as all samples are isolated (Figure 7). Compared to this, predispersion of the same amount of CNTs in THF for 60 min holds the maximum conductivity of 3.6 × 10^−4^ S/cm at 1 Hz. Hence, the use of surfactants leads to less stable dispersions with a high sedimentation and precipitation rate instead, where reagglomeration comes into effect immediately. This interferes with the formation of conductive paths. Furthermore, the presence of surfactant and solvent residues is allocated (see Figure 7b and Figure 8) which increases the distance between the carbon nanotubes and hinders the formation of electrical paths as well. In addition, surfactant residues reduce the agglomerate break-up due to shear forces during the mixing process, even though the concentration was limited to 0.1 wt.% and great effort was made regarding the removal of solvents and surfactants.

Stress–strain analyses likewise indicate a decrease in reinforcement implying deterioration in dispersion. Elongation at break values rise while the elastic modulus decreases at low strain. Surfactant and solvent residues account for the softening behavior, which is underlined by TEM images where surfactant residues can be observed in between CNT agglomerates (Figure 8). Compared to composites with CNT-predispersion solely in THF, pretreatment with SDBS in THF/H_2_O leads to an equivalent polymer–filler bonding apparent by the comparatively high tensile strength. Using “Brij35” in THF/H_2_O instead of SDBS, results in a material stiffness of the same level but decreased tensile strength and elongation at break implying impaired polymer–filler interactions.

As physical functionalization using surfactants did not enhance the electrical and mechanical performance, chemical surface modifications of CNTs were investigated, though this strongly affects the CNT structure and morphology. The chemical surface modification enables to equalize polarity differences between filler and polymer by adjusting the polarity of the filler surface and enhances polymer–filler interactions in consequence. Though, dealing with chemical functionalization implies the formation of covalent bonds between the carbon nanotube and the functionalization reagent which modifies the CNT structure. This obviously results in different CNT properties which turned out to diminish the electrical conductivity of CNTs due to tube damage and surface defects [41]. Therefore, it is essential to balance the benefit by increasing polymer–filler interactions and decreasing the conductivity. To determine best functionalization conditions, different oxidation methods were performed in this study (Table 1) as the oxidation is the most destroying but indispensable step for the following functionalization. An intermediate reduction process was applied here, which facilitates the formation of hydroxyl groups on the surface of CNTs. This is essential, as 7-octenyl-trichlorosilane is used for the following functionalization which owes a higher reactivity in the presence of hydroxyl groups (according to [35]). Table 1 displays the applied oxidation (Ox), reduction (Red), and functionalization (fCNT) methods in detail. The listed numbers concerning the reduction and functionalization step refer to the associated oxidation method.

**Figure 9:**
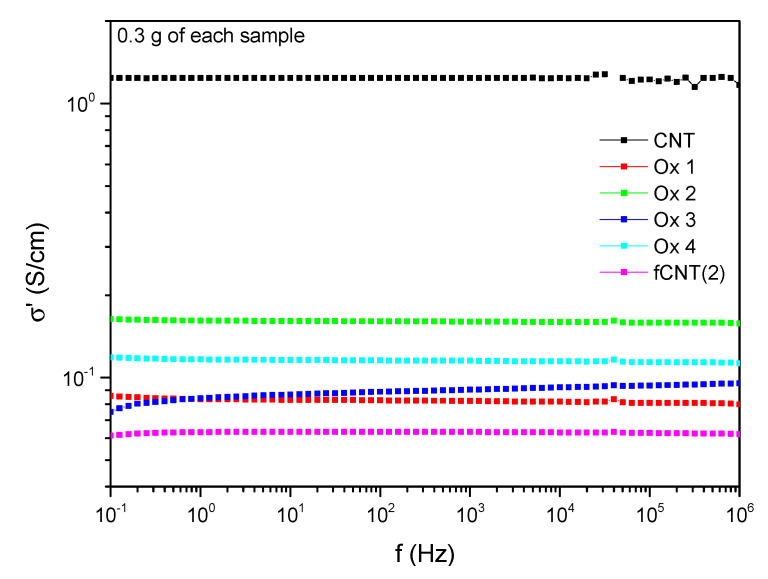
Intrinsic dielectric conductivity of modified and unmodified CNTs.

In order to reach best performance, the oxidation conditions were further modified based on the oxidation method, where best results concerning the electrical behavior are achieved (see Figure 9). As expected, this turned out to be at particular mild oxidation conditions (Ox 2). Details of the modification of “Ox 2” are shown in Table 2. Herein, temperature and duration of the oxidation process were varied.

It is known from literature, that the oxidation of graphene nanoplatelets to O/C-ratios in the range of 0.11 to 0.49 (corr. to C/O ratios of 1.01 to 2.0) has high influence e. g. on barrier properties and self-assembly behavior and only higher oxidation degrees yield self-standing structures [42]. This tendency is evident due to the change in polarity. Considering this, we can expect that on the one hand the more highly oxidized CNTs should be more difficult to be dispersed. On the other hand, concerning an appropriate balance between oxidation/functionalization-degree and the resulting polymer (PDMS)/CNT-interaction, we could expect an improvement in physical properties and electrical conductivity of the composites. Comparing the values of the XPS-measurements performed in this study the mildly oxidized types Ox 2_3 and Ox-2_5 in Table 2 are the most promising types.

According to the results of [41] a decrease in the electrical conductivity of at least one decade is observed which can be assigned to surface defects and the damage of the CNTs (Figure 9). Comparing the different oxidation conditions, “Ox 2” shows only marginal CNT damage ending up in the highest remaining conductivity of 1.6 × 10^−1^ S/cm at 1 Hz. Regarding the functionalized CNTs based on the mild oxidation method 2, the conductivity is further decreased resulting in a conductivity level less than all oxidized samples. This indicates an additional change of the CNT structure due to the subsequent processing step of the functionalization process.

**Figure 10:**
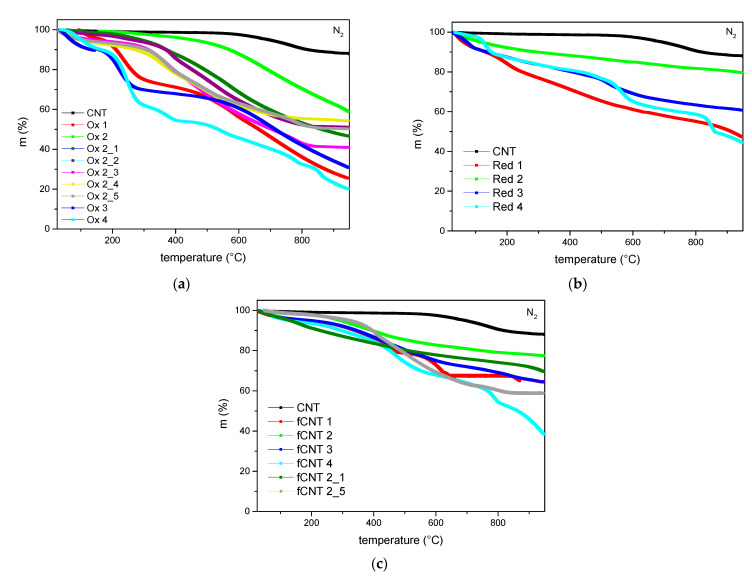
TG analyses of (**a**) oxidized CNTs, (**b**) reduced CNTs, and (**c**) functionalized CNTs.

As the functionalization steps definitely change the CNT structure, further filler characterization of the modified CNTs was performed to get an overview of their properties and potential. Thermal gravimetric analyses (Figure 10) represent the change in mass depending on the temperature with respect to functional groups on the surface of the carbon nanotubes. To prevent further oxidation, N_2_ remained the carrier gas for the whole experiment.

The curve characteristics and a relatively high remaining mass of 59% of the CNTs oxidized using method 2 confirm the lowest structural change by this modification process, whereas a higher number of functional groups on the surface can be observed for oxidation method 1, 3, and 4 (Figure 10a). The latter ones also represent a high loss of mass at relatively low temperatures which can be assigned to adsorbed molecules like oxygen. Comparing the oxidation conditions that varied within the series of oxidation method 2, the initial parameters using a reaction temperature of 75 °C for 180 min preserve the surface structure of the carbon nanotubes best. The modification toward milder conditions results in an increased incineration throughout the thermal gravimetric experiment indicating inadequate reaction conditions during the oxidation process and an increase in functional groups on the CNT surface instead. Further processed samples like reduced and functionalized CNTs (Figure 10b,c) predominately show the same progression with regard to their base oxidation method. Overall, the results confirm that applying oxidation method 2 for further processing leads to the slightest structural change and is therefore preferred.

Raman spectroscopy was performed in order to investigate the structural defects on the CNT surface due to the modification processes as it is extremely sensitive toward changes in morphology. Raman-bands of the investigated samples were assigned to the graphitic structure as CNTs consist of rolled up graphene sheets. Therefore, the *G*-band at around 1590 cm^−1^ (planar vibration of sp^2^-carbon atoms) and the *D*-band at around 1350 cm^−1^ (sp^3^, disorder of graphene structure) are appropriate bands to investigate graphitic defect structures. The intensity ratio I*_D_*/I*_G_* states the extent of defects, where I*_D_*/I*_G_* increases with rising defects. An additional evidence on structural defects can be found regarding the *G′*-band at around 2650 cm^−1^, which represents the second-order process of the sp^2^-vibration.

**Figure 11:**
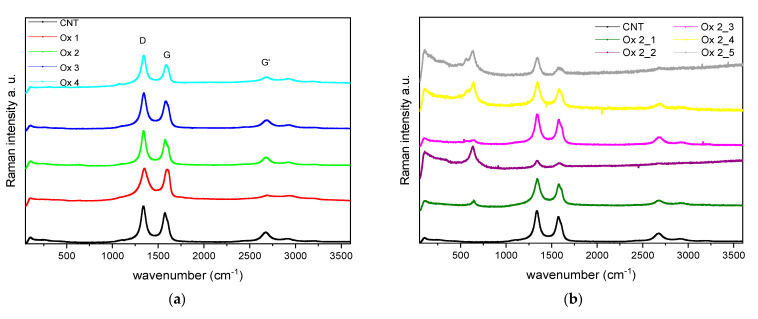

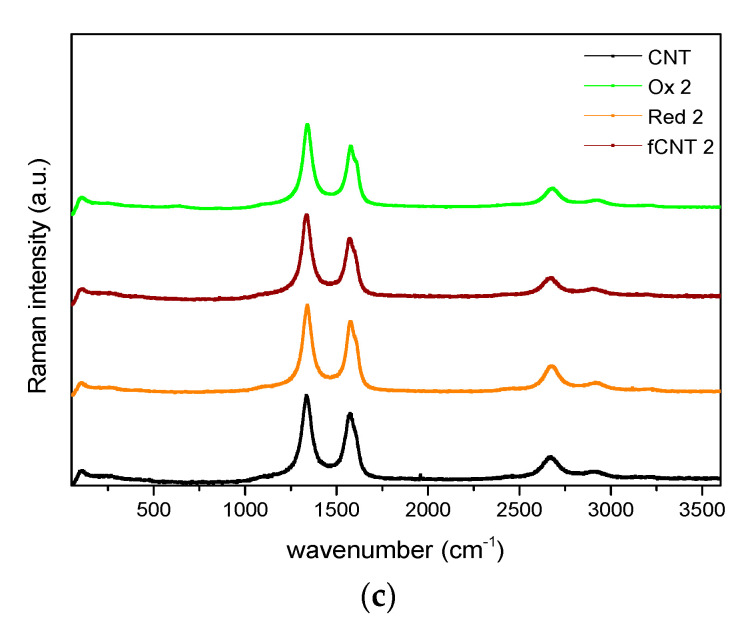
Raman-spectra of (**a**) oxidized, (**b**) reduced, and (**c**) functionalized CNTs.

Figure 11 represents the Raman-intensities against the wavelength. According to further processing, the ratio *I_D_/I_G_* and therewith surface defects increase in the order of pristine CNTs < oxidation < reduction < functionalization (Figure 11c and Table 3). This is due to the conversion of sp^2^-hybridized carbon atoms (C=C) into sp^3^-hybridized ones. Comparing the different oxidation methods (Figure 11a), the lowest defect quantity is observed for the oxidation method “Ox 1,” which is even lower than those of the pristine carbon nanotubes. As the *G′*-band in this spectrum has experienced a severe decrease, this effect is dominated by the purification due to the acid based oxidation method and does not represent the defect level. Consequently, oxidation method 2 and 3 reveal the lowest defect quantity as the *I_D_/I_G_*-ratio is low and the *G′*-band is comparatively unaffected in addition.

Regarding the differences by varying the oxidation conditions of method 2 (Figure 11b), improper conditions are confirmed again. Even though the intensity ratio *I_D_/I_G_* and calculation of aromatic cluster size implies the oxidation methods “Ox 2_1” up to “Ox 2_4” to be appropriate (apparent by low *I_D_/I_G_*-values), an additional Raman-band at around 500 cm^−1^ arises in the spectra of these samples. As this band can be assigned to amorphous sp^3^-carbon atoms this shows that merely the processing belonging to “Ox 2_3” performs satisfactorily. Overall, many structural defects are observed applying method “Ox 2_5.”

To further analyze the surface chemistry of pretreated CNTs regarding the type of functional groups, X-ray photoelectron spectroscopy measurements (XPS) were performed. Hereby, the CNTs are irradiated with X-rays under ultra-high vacuum conditions and the emitted characteristic photoelectrons are detected regarding their quantity and kinetic energy. The later one enables to obtain binding energies and consequently gives evidence on the types of functional groups. As the penetration depth amounts to maximum 20 nm, this ensures to merely analyze surface elements.

**Figure 12:**
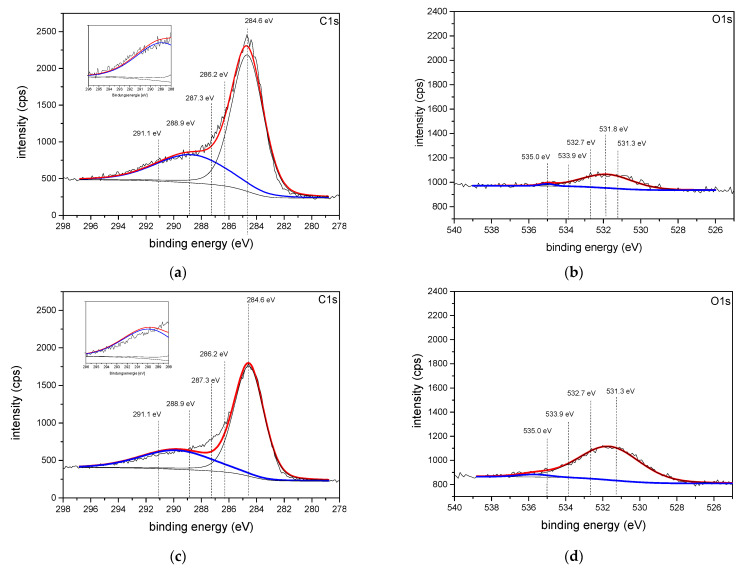

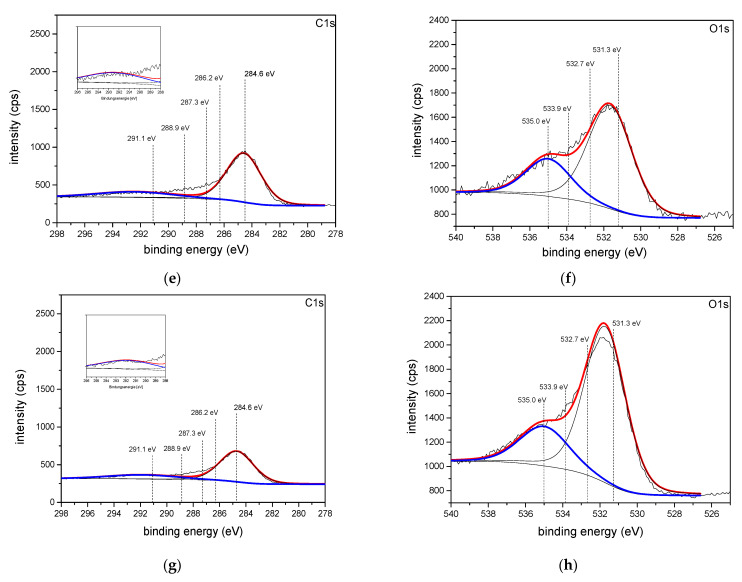
XPS analyses of carbon (C1s) and oxygen (O1s) areas of CNTs, (**a**) + (**b**) pristine CNTs, (**c**) + (**d**) oxidized according to “Ox 2”, (**e**) + (**f**) oxidized according to “Ox 2_3” and (**g**) + (**h**) oxidized according to “Ox 2_5”.

Selected oxidized CNTs (oxidized using method 2, 2_3 and 2_5) were analyzed and compared to pristine carbon nanotubes using XPS (Figure 12). As the emitted signals emerge in different spectroscopic areas, a differentiation between carbon, covalent-bonded oxygen, and adsorbed ions due to the oxidation treatment is practicable. The carbon signal (C1s) can be interpreted following the investigations of Sun et al. [42], which are based on graphene-oxide. According to this, the signal at 284.6 eV refers to sp^2^-hybridized carbon and is used for the calibration, the signal at 286.2 eV is referred to C–O–bondings of hydroxyl-, epoxy-, and phenol groups, the signal at 287.3 eV is correlated to C=O–bondings of keto- and aldehyde-groups, and the peak at 288.9 eV corresponds to O–C=O-bondings of carboxy- and ester-groups. In addition, the signal at binding energies of 291.1 eV is referred to π-π*-signals of sp^2^−atoms which decreases at mild oxidation conditions (Ox 2_3 and 2_5) due to the degradation of sp^2^−hybridized carbon atoms. This supports the thermal gravimetric results indicating that the oxidation reaction at mild conditions is improper resulting in an increase of oxygen-functionalized carbon nanotubes [43]. Regarding the oxygen signals (O1s) an increase in intensity and therefore in oxygen concentration on the surface of the carbon nanotubes can be observed by the oxidation process and further on due to mild and improper oxidation. The signal correlation was performed according to Dongil et al. [44] where the signal at 531.8 eV consists of three main peaks: O=C-bondings of keto and aldehyde groups at 531.3 eV, O–C-bondings of epoxy- and phenolgroups at 532.7 eV, and carboxylic acid- and ester-functionalities at 533.9 eV. The minor signal at 535 eV of pristine CNTs can be referred to adsorbed water.

Table 4 contains further evaluations of XPS measurements concerning the proportional amounts of oxygen and carbon of the CNTs. As reported before, the oxidation of CNTs increases the amount of oxygen on the CNT surface due to the formation of functional groups resulting in a decrease of the C/O–ratio. This trend is extremely significant for the mild oxidation conditions due to an additional formation of functional groups. In accordance with Wepasnick et al. [33] there is no distinct variation in the distribution of the oxygenic functional groups, as all samples analyzed by XPS were exposed to the same oxidizing agent merely varying the oxidation conditions.

**Figure 13:**
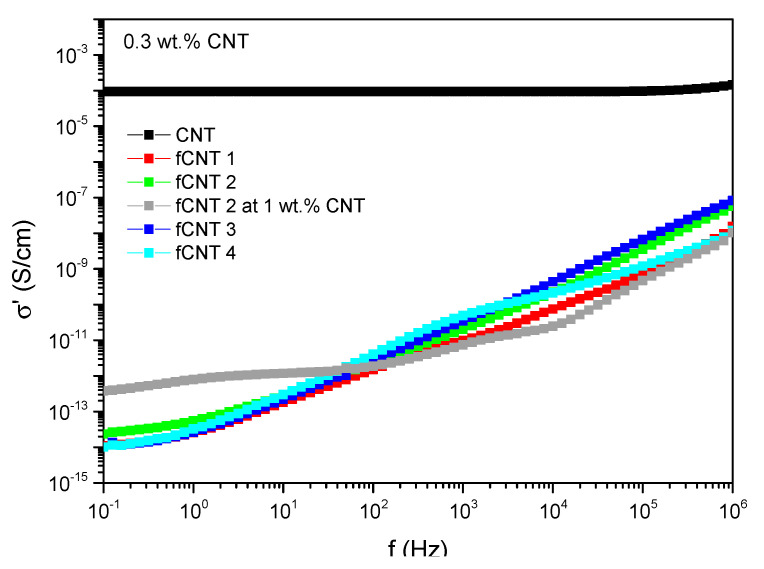
Electrical conductivity of PDMS compounds with functionalized CNTs.

Regarding the incorporation of the chemically modified CNTs into the PDMS matrix, 0.3 wt.% of functionalized CNTs were pre-dispersed for 60 min by ultrasonication in THF, as these pre-dispersion conditions turned out to be further promising. Analyzing the electrical conductivity, 0.3 wt.% of pre-dispersed CNTs without any functionalization already result in a conductive material with a conductivity of 9.4 × 10^−5^ S/cm whereas all samples with functionalized CNTs are insulating and indicate non-percolated systems (Figure 13). Even those compounds where the functionalization was based on oxidation method 2, which preserves the CNT structure best and resulted in the highest remaining electrical conductivity (see Figure 9) are insulating. Increasing the amount of functionalized CNTs up to 1 wt.% does not increase the intrinsic conductivity substantial. Here, just a minimal conductivity of 7.9 × 10^−13^ S/cm at 1 Hz is obtained for samples filled with 1 wt.% fCNT 2.

According to the characterization of the modified CNTs, the chemical modification processes change the aromatic CNT structure where the sp^2^-hybridized carbon atoms are converted into sp^3^-ones. As π-electrons are inevitable for the conduction mechanism, the functional groups on the CNT surface increase the electrical resistance due to the formation of sp^3^-hybrids [8]. Consequently, the sp^3^-defects decrease not only the electrical conductivity level of the modified carbon nanotubes (Figure 9) but also lead to non-conducting CNT/PDMS composites. Additional CNT fracture reduces the tube length which hinders the formation of an electrical filler network and further decreases the conductivity.

**Figure 14:**
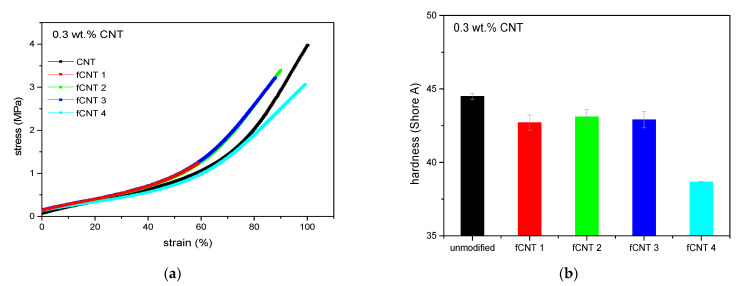
Mechanical properties depending on the functionalization method (**a**) stress–strain behavior and (**b**) Shore A hardness.

The approach of the functionalization step was to increase the compatibility between filler and polymer and therefore the polymer–filler interactions to simultaneously decrease the filler–filler interactions and to increase the filler dispersion by this. As the conductivity level is extremely reduced in functionalized CNT/PDMS composites, a potential benefit in dispersion cannot be observed here and the effect is superimposed by the decrease in conductivity due to CNT defects. Hence, mechanical analyses were performed to examine a potential increase in polymer–filler interactions due to the functionalization process (Figure 14).

**Figure 15:**
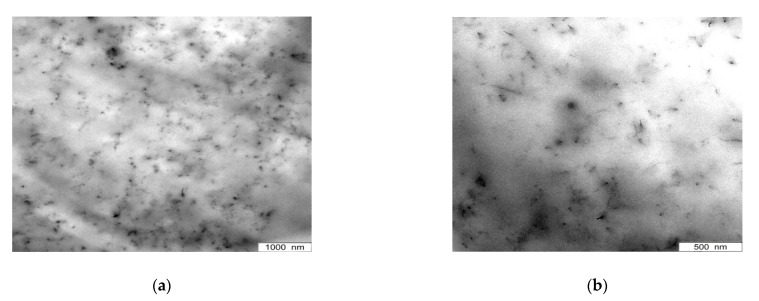
TEM images of PDMS/CNT-composites with CNTs oxidized using method “Ox 2” ((**a**,**b**), two different scales).

Compared to CNT/PDMS-composites where merely the pre-dispersion step was performed, a decrease in tensile strength and elongation at break can be seen for all functionalized samples, especially those based on oxidation method 1. Regarding stress–strain curves and hardness measurements, oxidation methods 2 and 3 taken as basic strategy for the functionalization result in a comparatively little decrease in the mechanical performance. This is in good accordance with the results obtained by Raman spectroscopy. Though, no enhancement in reinforcement or hardness can be observed due to functionalization. This is contrary to the expectations since the modification of the CNTs was performed to improve the compatibility between filler and polymer by reversing the polarity of the CNT surface due to the application of alkyl chains [35]. In addition to the change in polarity, the terminal double bond of the alkyl chains enables to form covalent bonds to the polymer during the addition cured vulcanization process which further enhances the bonding of the CNTs to the PDMS. Therefore, CNT damage is likely to occur which was assumed regarding the electrical conductivity results already. To consider this in detail, TEM images were recorded to have a microscopic insight in the nanoscale. Hereby, a reduction in tube lengths can be seen in CNT/PDMS-composites where the CNTs were oxidized using method “Ox 2” (Figure 15) compared to composites where simply pre-dispersion in THF was performed (Figure 3d). The aspect ratio decreases due to the oxidation process which proves the assumption of CNT damage. Besides this, good dispersion with only minor CNT-clusters can be observed here verifying agglomerate break-up. Therefore, reduced filler–filler interactions and enhanced polymer–filler interactions are indicated which are superimposed by CNT damage. Finally, the functionalization result in a decrease in electrical and mechanical performance of the composite as the CNT damage is the predominant effect.

## 4. Discussion and Conclusions

A break-up of CNT-agglomerates by CNT pretreatment can be realized using a pre-dispersion step before the mixing process. Hereby, the carbon nanotubes are separated by the solvent and the filler–filler interactions are decreased. This ends up in improved filler dispersion and a distinct increase in electrical conductivity. Due to the high filler dispersion, a conductive filler network is built up at low CNT amounts and the percolation threshold is decreased to 0.1 wt.% of CNTs. Best results are obtained using an ultrasonic finger for 60 min in THF, where an electrical conductivity of 3.2 × 10^−4^ S/cm at 1 Hz is reached at 0.5 wt.% of CNTs. Regarding the solvent performance, the tendency THF > acetone > chloroform corresponding to their eluting order is observed regarding dielectric results. This implies an additional contribution to the electrical conductivity due to solvent residues which is confirmed by mechanical analyses. A softening effect is determined here, apparent by a distinct deterioration of the elongation at break and Shore A hardness. In addition, enhanced dispersion is observed which partly compensates the softening effect due to solvent residues.

Adding oleic acid results in a further increase in electrical conductivity of minor extend. As the oleic acid covers the surface of the carbon nanotubes, solvent adhesion is inhibited, and no softening effect can be observed. Though, there is no mechanical reinforcement. Besides this, the use of the surfactants SDBS and “Brij 35” for physical functionalization leads to no improvement, neither in electrical conductivity nor in mechanical properties. Surfactant residues cause CNT re-agglomeration here, impairing the dispersion and subsequently resulting in electrical isolating composites.

Chemical functionalization of the carbon nanotubes modifies the CNT surface and enhances the compatibility of PDMS and CNTs. This leads to an increase in CNT dispersion due to enhanced polymer–filler interactions and reduced filler–filler interactions. Though, the oxidation process damages the carbon nanotubes resulting in CNT fracture. Finally, the potential improvement in electrical conductivity and reinforcement due to the improved dispersion is superimposed by the CNT damage. Especially the oxidation process holds a high impact on the conductivity, resulting in a decrease for at least one decade even at mild oxidation conditions. During the oxidation process, sp^2^-hybridized carbon atoms are converted into sp^3^ ones which decrease the electrical conductivity as π-electrons are inevitable for the conductivity. Overall, using mild oxidation conditions preserves structural integrity best, whereas moderating the oxidation conditions even milder leads to inadequate reaction conditions resulting in propagated functional groups and impeded formation of electrical pathways. Any subsequent functionalization steps cause additional changes in the CNT structure and further impair the electrical conductivity.

## Figures and Tables

**Table 1:** Applied oxidation, reduction, and functionalization steps in detail.

Name	Chemicals	Temperature (°C)	Duration (min)	Reference
Ox 1	220 mmol concentrated H_2_SO_4_, 3 mmol NaNO_3_, 9.5 mmol KMnO_4_ (97%)	80	20	Pazat [31]
Ox 2	0.1 M KMnO_4_−solution	75	180	Malikov et al. [32]
Ox 3	0.5 M H_2_SO_4_−solution, 0.47 mmol KMnO_4_ (97%)	130	240	Wepasnick et al. [33]
Ox 4	9 M H_2_SO_4_−solution, 1.3 mmol KMnO_4_ (97%)	25	360	Araújo et al. [34]
Red no.	0.28 mmol DIBAL-H, toluene	25	240	Vast et al. [35]
fCNT no.	0.33 mmol Et_3_N, 0.01 M solution of 7-octenyl-trichlorosilane in toluene (N_2_-Inertgas)	25	1440	Vast et al. [35]

**Table 2:** Further modification of oxidation method 2 (Ox 2).

Name	Temperature (°C)	Duration (min)
Ox 2_1	75	30
Ox 2_2	25	180
Ox 2_3	25	30
Ox 2_4	75	5
Ox 2_5	25	5

**Table 3:** Ratio of Raman-intensities *I_D_/I_G_* and aromatic cluster size.

Sample	*I_D_/I_G_*	Aromatic Cluster Size
CNT	1.20	17.15
Ox 1	1.03	19.74
Ox 2	1.29	16.68
Ox 2_1	1.22	17.04
Ox 2_2	1.15	17.69
Ox 2_3	1.21	17.10
Ox 2_4	1.28	16.75
Ox 2_5	1.63	14.13
Red 2	1.32	16.33
fCNT 2	1.36	16.18
Ox 3	1.31	16.35
Ox 4	1.33	16.25

**Table 4:** XPS results referred to the amounts of carbon and oxygen.

Sample	C1s (%)	O1s (%)	C/O-Ratio
CNT	99.8	1.6	62.4
Ox 2	93.5	6.4	14.6
Ox 2_3	69.4	30.5	2.3
Ox 2_5	54.1	45.8	1.2

## Data Availability

Not applicable.
